# *De novo* gene birth

**DOI:** 10.1371/journal.pgen.1008160

**Published:** 2019-05-23

**Authors:** Stephen Branden Van Oss, Anne-Ruxandra Carvunis

**Affiliations:** Department of Computational and Systems Biology, Pittsburgh Center for Evolutionary Biology and Medicine, School of Medicine, University of Pittsburgh, Pittsburgh, PA, United States of America

***De novo* gene birth** is the process by which new genes evolve from DNA sequences that were ancestrally non-genic. *De novo* genes represent a subset of novel genes, and may be protein-coding or instead act as RNA genes [1]. The processes that govern *de novo* gene birth (**Fig 1A**) are not well understood, though several models exist that describe possible mechanisms by which *de novo* gene birth may occur. Although *de novo* gene birth may have occurred at any point in an organism’s evolutionary history, ancient *de novo* gene birth events are difficult to detect. Most studies of *de novo* genes to date have thus focused on young genes, typically taxonomically-restricted genes (TRGs) that are present in a single species or lineage, including so-called orphan genes, defined as genes that lack any identifiable homolog. It is important to note, however, that not all orphan genes arise *de novo*, and instead may emerge through fairly well-characterized mechanisms such as gene duplication (including retroposition) or horizontal gene transfer followed by sequence divergence, or by gene fission/fusion [2, 3] (**Fig 2**) Though *de novo* gene birth was once viewed as a highly unlikely occurrence [4], there are now several unequivocal examples of the phenomenon that have been described. It furthermore has been advanced that *de novo* gene birth plays a major role in the generation of evolutionary innovation [5, 6].

**Fig 1:**
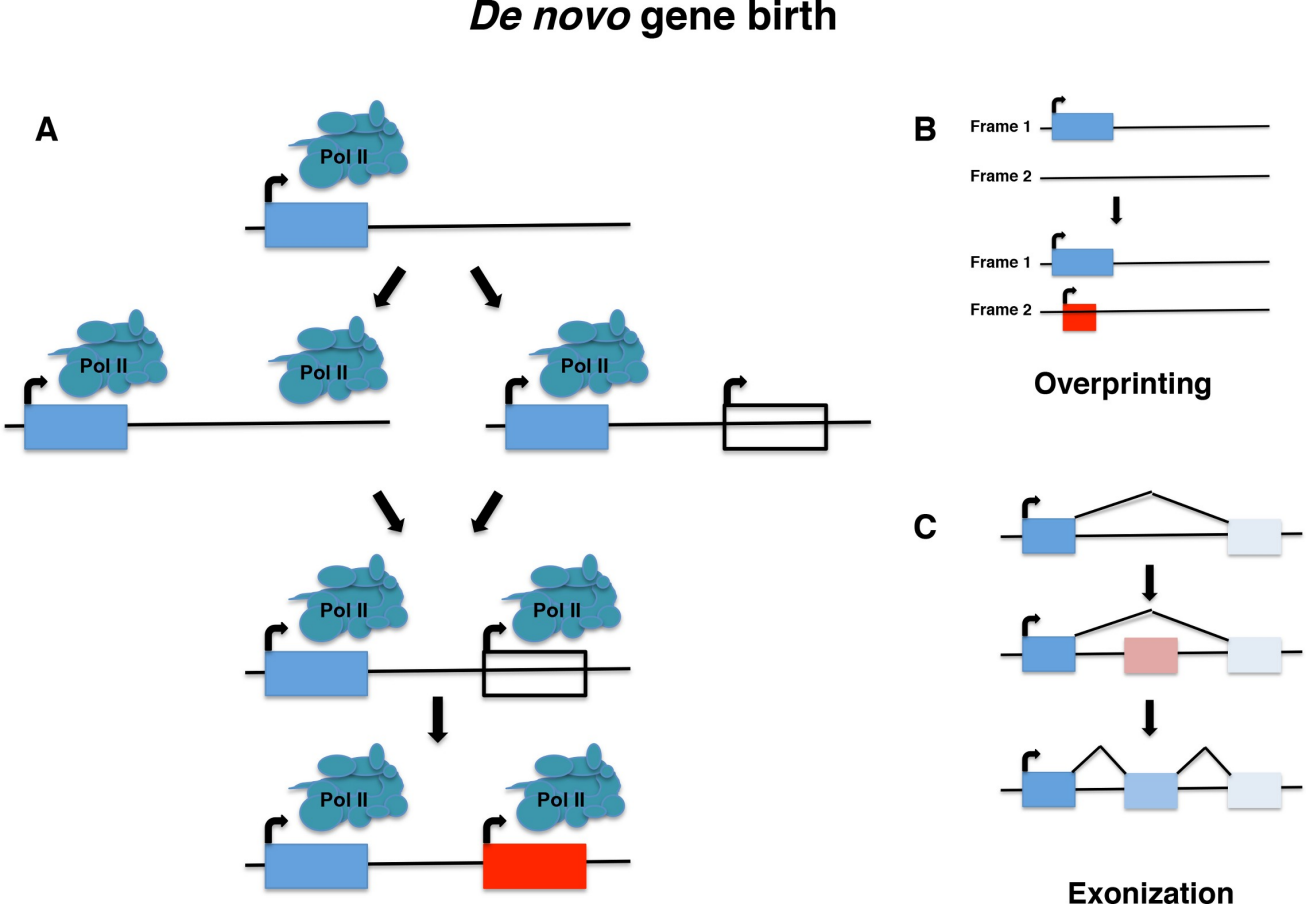
*De novo* gene birth. Novel genes can emerge from ancestrally non-genic regions through poorly understood mechanisms. **(A)** A non-genic region first gains transcription and an ORF, in either order, facilitating the birth of a *de novo* gene. The ORF is for illustrative purposes only, as *de novo* genes may also be multi-exonic, or lack an ORF, as with RNA genes. **(B)** Overprinting. A novel ORF is created that overlaps with an existing ORF, but in a different frame. **(C)** Exonization. A formerly intronic region becomes alternatively spliced as an exon, such as when repetitive sequences are acquired through retroposition and new splice sites are created through mutational processes. Overprinting and exonization may be considered as special cases of *de novo* gene birth.

**Fig 2:**
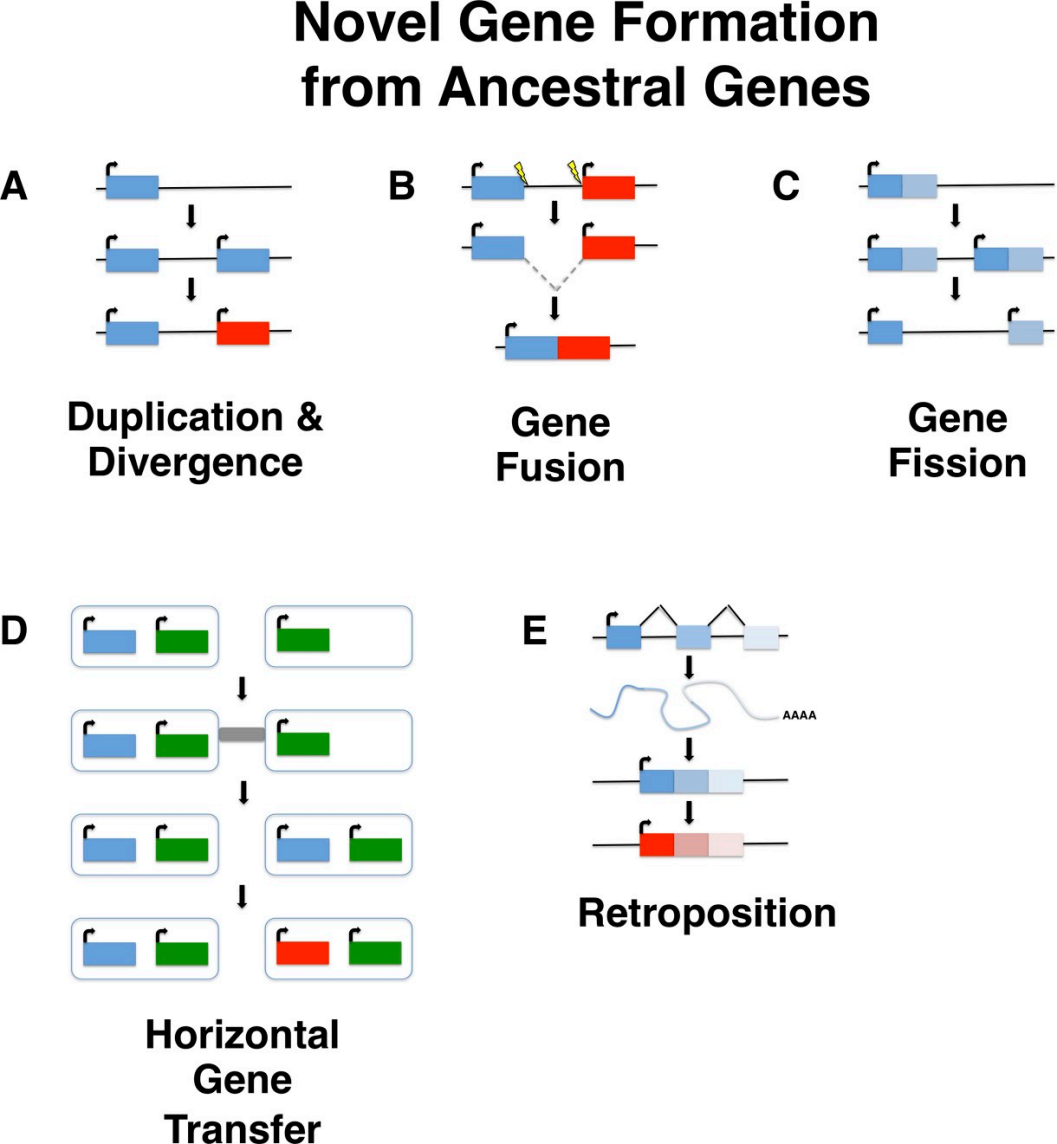
Novel gene formation from ancestral genes. Novel genes can be formed from ancestral genes through a variety of mechanisms. Inspired by Table 1 from [7]. **(A)** Duplication and divergence. Following duplication, one copy experiences relaxed selection and gradually acquires novel function(s). **(B)** Gene fusion. A hybrid gene formed from some or all of two previously separate genes. Gene fusions can occur by different mechanisms; shown here is an interstitial deletion. **(C)** Gene fission. A single gene separates to form two distinct genes, such as by duplication and differential degeneration of the two copies [8]. **(D)** Horizontal gene transfer. Genes acquired from other species by horizontal transfer undergo divergence and neofunctionalization. **(E)** Retroposition. Transcripts may be reverse transcribed and integrated as an intronless gene elsewhere in the genome. This new gene may then undero divergence.

## 1 History of the study of *de novo* gene birth

As early as the 1930s, J.B.S. Haldane and others suggested that copies of existing genes may lead to new genes with novel functions [3]. In 1970, Susumu Ohno published the seminal text *Evolution by Gene Duplication [9]*. For some time subsequently, the consensus view was that virtually all genes were derived from ancestral genes [10], with François Jacob famously remarking in a 1977 essay that “the probability that a functional protein would appear *de novo* by random association of amino acids is practically zero” [4]. In the same year, however, Pierre-Paul Grassé coined the term “overprinting” to describe the emergence of genes through the expression of alternative open reading frames (ORFs) that overlap preexisting genes [11] (**Fig 1B)**. These new ORFs may be out of frame with or antisense to the preexisting gene. They may also be in frame with the existing ORF, creating a truncated version of the original gene, or represent 3’ extensions of an existing ORF into a nearby ORF. The first two types of overprinting may be thought of as a particular subtype of *de novo* gene birth; although overlapping with a previously coding region of the genome, the primary amino-acid sequence of the newly encoded protein is entirely novel. The first examples of this phenomenon in bacteriophages were reported in a series of studies from 1976 to 1978 [12–14], and since then numerous other examples have been identified in viruses, bacteria, and several eukaryotic species [15–19]. The phenomenon of exonization also represents a special case of *de novo* gene birth, in which, for example, often-repetitive intronic sequences acquire splice sites through mutation, leading to *de novo* exons (**Fig 1C**). This was first described in 1994 in the context of *Alu* sequences found in the coding regions of primate mRNAs [20]. Interestingly, such *de novo* exons are frequently found in minor splice variants, which may allow the evolutionary “testing” of novel sequences while retaining the functionality of the major splice variant(s) [21].

Still, it was thought by some that most or all eukaryotic proteins were constructed from a constrained pool of “starter type” exons [22]. Using the sequence data available at the time, a 1991 review estimated the number of unique, ancestral eukaryotic exons to be < 60,000 [22], while in 1992 a piece was published estimating that the vast majority of proteins belonged to no more than 1,000 families [23]. Around the same time, however, the sequence of chromosome III of the budding yeast *Saccharomyces cerevisiae* was released [24], representing the first time an entire chromosome from any eukaryotic organism had been sequenced. Sequencing of the entire yeast nuclear genome was then completed by early 1996 through a massive, collaborative international effort [25]. In his review of the yeast genome project, Bernard Dujon noted that the unexpected abundance of genes lacking any known homologs was perhaps the most striking finding of the entire project [25].

In 2006 and 2007, a series of studies provided arguably the first documented examples of full-length *de novo* gene birth [26–28]. An analysis of the accessory gland transcriptomes of *Drosophila yakuba* and *Drosophila erecta* first identified 20 putative lineage-restricted genes that appeared unlikely to have resulted from gene duplication [28]. Levine and colleagues then confirmed the *de novo* origination of five genes specific to *Drosophila* melanogaster and/or the closely related *Drosophila simulans* through a rigorous pipeline that combined bioinformatic and experimental techniques [27]. These genes were identified by combining BLAST search-based and synteny-based approaches (see below), which demonstrated the absence of the genes in closely-related species [27]. Despite their recent evolution, all five genes appear fixed in *D*. *melanogaster*, and the presence of paralogous non-coding sequences that are absent in close relatives suggests that four of the five genes may have arisen through a recent intrachromosomal duplication event *[27]*. Interestingly, all five were preferentially expressed in the testes of male flies [27] (see below). The three genes for which complete ORFs exist in both *D*. *melanogaster* and *D*. *simulans* showed evidence of rapid evolution and positive selection [27]. This is consistent with a recent emergence of these genes, as it is typical for young, novel genes to undergo adaptive evolution [29–31]. A subsequent study using methods similar to Levine *et al*. and an expressed sequence tag library derived from *D*. *yakuba* testes identified seven genes derived from six unique *de novo* gene birth events in *D*. *yakuba* and/or the closely related *D*. *erecta* [26]. Three of these genes are extremely short (<90 bp), suggesting that they may be RNA genes [26], although several examples of very short functional peptides have also been documented [32–35]. Around the same time as these studies in *Drosophila* were published, a homology search of genomes from all domains of life, including 18 fungal genomes, identified 132 fungal-specific proteins, 99 of which were unique to *S*. *cerevisiae* [36].

Since these initial studies, many groups have identified specific cases of *de novo* gene birth events in diverse organisms [37]. The *BSC4* gene in *S*. *cerevisiae*, identified in 2008, shows evidence of purifying selection, is expressed at both the mRNA and protein levels, and when deleted is synthetically lethal with two other yeast genes, all of which indicate a functional role for the *BSC4* gene product [38]. Historically, one argument against the notion of widespread *de novo* gene birth is the evolved complexity of protein folding. Interestingly, Bsc4 was later shown to adopt a partially folded state that combines properties of native and non-native protein folding [39]. Another well-characterized example in yeast is *MDF1*, which both represses mating efficiency and promotes vegetative growth, and is intricately regulated by a conserved antisense ORF [40, 41]. In plants, the first *de novo* gene to be functionally characterized was *QQS*, an *Arabidopsis thaliana* gene identified in 2009 that regulates carbon and nitrogen metabolism [42]. The first functionally characterized *de novo* gene identified in mice, a noncoding RNA gene, was also described in 2009 [43]. In primates, a 2008 informatic analysis estimated that 15/270 primate orphan genes had been formed *de novo [44]*. A 2009 report identified the first three *de novo* human genes, one of which is a therapeutic target in chronic lymphocytic leukemia [45]. Since this time, a plethora of genome-level studies have identified large numbers of orphan genes in many organisms (**Table 1**), although the extent to which they arose *de novo* remains debated.

**Table 1:** Genome-scale studies of orphan and *de novo* genes in various lineages. For purposes of this table, genes are defined as **orphan genes** (when species-specific) or **TRGs** (when limited to a closely related group of species) when the mechanism of origination has not been investigated, and as ***de novo* genes** when *de novo* origination has been inferred, irrespective of method of inference. The designation of *de novo* genes as “candidates” or “proto-genes” reflects the language used by the authors of the respective studies.

Organism /Lineage	Homology Detection Method(s)	Evidence of Expression?	Evidence of Selection?	Evidence of Physiological Role?	# Orphan/*De Novo* Genes	Notes	Ref.
Arthropods	BLASTP for all 30 species against each other, TBLASTN for *Formicidae* only, searched by synteny for unannotated orthologs in *Formicidae* only	ESTs, RNA-seq; RT-PCR on select candidates	37 *Formicidae*-restricted orthologs appear under positive selection (M1a to M2a and M7 to M8 models using likelihood ratio tests); as a group, *Formicidae*-restricted orthologs have a significantly higher K_a_/K_s_rate than non-restricted orthologs	Prediction of signal peptides and subcellular localization for subset of orphans	~65,000 orphan genes across 30 species	Abundance of orphan genes dependent on time since emergence from common ancestor; >40% of orphans from intergenic matches indicating possible *de novo* origin	[80]
*Arabidopsis thaliana*	BLASTP against 62 species, PSI-BLAST against NCBI nonredundant protein database, TBLASTN against PlantGDB-assembled unique transcripts database; searched syntenic region of two closely related species	Transcriptomic and translatomic data from multiple sources	Allele frequencies of *de novo* genes correlated with their DNA methylation levels	None	782 *de novo* genes	Also assessed DNA methylation and histone modifications	[62]
*Bombyx mori*	BLASTP against four lepidopterans, TBLASTN against lepidopteran EST sequences, BLASTP against NCBI nonredundant protein database	Microarray, RT-PCR	None	RNAi on five *de novo* genes produced no visible phenotypes	738 orphan genes	Five orphans identified as *de novo* genes	[87]
*Brassicaceae*	BLASTP against NCBI nonredundant protein database, TBLASTN against NCBI nucleotide database, TBLASTN against NCBI EST database, PSI-BLAST against NCBI nonredundant protein database, InterProScan [145]	Microarray	None	TRGs enriched for expression changes in response to abiotic stresses compared to other genes	1761 nuclear TRGs; 28 mitochondrial TRGs	~2% of TRGs thought to be *de novo* genes	[88]
*Drosophila melanogaster*	BLASTN of query cDNAs against *D*. *melanogaster*, *D*. *simulans* and *D*. *yakuba* genomes; also performed check of syntenic region in sister species	cDNA/ expressed sequence tags (ESTs)	K_a_/K_s_ ratios calculated between retained new genes and their parental genes are significantly >1, indicating most new genes are functionally constrained	List includes several genes with characterized molecular roles	72 orphan genes; 2 *de novo* genes	Gene duplication dominant mechanism for new genes; 7/59 orphans specific to *D*. *melanogaster* species complex identified as *de novo*	[65]
*Drosophila melanogaster*	Presence or absence of orthologs in other *Drosophila* species inferred by synteny based on UCSC genome alignments and FlyBase protein-based synteny; TBLASTN against *Drosophila* subgroup	Indirect (RNAi)	Youngest essential genes show signatures of positive selection (α = 0.25 as a group)	Knockdown with constitutive RNAi lethal for 59 TRGs	195 “young” (>35myo) TRGs; 16 *de novo* genes	Gene duplication dominant mechanism for new genes	[63]
*Drosophila melanogaster*	RNA-seq in *D*. *melanogaster* and close relatives; syntenic alignments with *D*. *simulans* and *D*. *yakuba*; BLASTP against NCBI nonredundant protein database	RNA-seq	Nucleotide diversity lower in non-expressing relatives; Hudson-Kreitman-Aguade-like statistic lower in fixed *de novo* genes than in intergenic regions	Structural features of *de novo* genes (e.g. enrichment of long ORFs) suggestive of function	106 fixed and 142 segregating *de novo* genes	Specifically expressed in testes	[64]
*Homo sapiens*	BLASTP against other primates; BLAT against chimpanzee and orangutan genomes, manual check of syntenic regions in chimpanzee and orangutan	RNA-seq	Substitution rate provides some evidence for weak selection; 59/60 *de novo* genes are fixed	None	60 *de novo* genes	Enabling mutations identified; highest expression seen in brain and testes	[66]
*Homo sapiens*	BLASTP against chimpanzee, BLAT and Ssearch of syntenic region in chimpanzee, manual check of syntenic regions in chimpanzee and macaque	EST/cDNA	No evidence of selective constraint seen by nucleotide divergence	One of the genes identified has a known role in leukemia	3 *de novo* genes	Estimated that human genome contains ~ 18 human-specific *de novo* genes	[45]
*Lachancea* and *Saccharomyces*	BLASTP of all focal species against each other, BLASTP against NCBI nonredundant protein database, PSI-BLAST against NCBI nonredundant protein database, HMM Profile-Profile of TRG families against each other; families then merged and searched against four profile databases	Mass Spectrometry (MS)	K_a_/K_s_ ratios across *Saccharomyces*indicate that candidates are under weak selection that increases with gene age; in *Lachancea* species with multiple strains, pN/pS ratios are lower for *de novo* candidates than for "spurious TRGs"	None	288 candidate *de novo* genes	MS evidence of translation for 25 candidates	[90]
*Mus musculus* and *Rattus norvegicus*	BLASTP of rat and mouse against each other, BLASTP against Ensembl compara database; searched syntenic regions in rat and mouse	UniGene Database	Subset of genes shows low nucleotide diversity and high ORF conservation across 17 strains	Two mouse genes cause morbidity when knocked out	69 *de novo* genes in mouse and 6 "de novo" genes in ra	Enabling mutations identified for 9 mouse genes	[146]
*Mus musculus*	BLASTP against NCBI nonredundant protein database	Microarray	None	None	781 orphan genes	Age-dependent features of genes compatible with *de novo* emergence of many orphans	[76]
*Oryza*	Protein-to-protein and nucleotide-to-nucleotide BLAT against eight *Oryza* species and two outgroup species; searched syntenic regions of these species for coding potential	RNA-seq (all *de novo* TRGs); Ribosome Profiling and targeted MS (some *de novo* TRGs)	22 *de novo* candidates appear under negative selection, and six under positive selection, as measured by K_a_/K_s_ rate	Expression of *de novo* TRGs is tissue-specific	175 *de novo* TRGs	~57% of *de novo* genes have translational evidence; transcription predates coding potential in most cases	[147]
Primates	BLASTP against 15 eukaryotes, BLASTN against human genome, analysis of syntenic regions	ESTs	K_a_/K_s_ ratios for TRGs below one but higher than established genes; coding scores consistent with translated proteins	Several genes have well-characterized cellular roles	270 TRGs	~5.5% of TRGs estimated to have originated *de novo*	[44]
*Rodentia*	BLASTP against NCBI nonredundant protein database	None	Mouse genes share 50% identity with rat ortholog	None	84 TRGs	Species-specific genes excluded from analysis; results robust to evolutionary rate	[98]
*Saccharomyces cerevisiae*	BLASTP and PSI-BLAST against 18 fungal species, HMMER and HHpred against several databases, TBLASTN against three close relatives	None	None	Majority of orphans have characterized fitness effects	188 orphan genes	Ages of genes determined at level of individual residues	[83]
*Saccharomyces cerevisiae*	BLASTP, TBLASTX, and TBLASTN against 14 other yeast species, BLASTP against NCBI nonredundant protein database	Ribosome Profiling	All 25 *de novo* genes, 115 proto-genes under purifying selection (pN/pS < 1)	None	25 *de novo* genes; 1,891 “proto-genes”	*De novo* gene birth more common than new genes from duplication; proto-genes are unique to *Saccbaromyces sensu strictu* yeasts	[75]
*Saccharomyces sensu strictu*	BLASTP against NCBI nonredundant protein database, TBLASTN against ten outgroup species; BLASTP and phmmer against 20 yeast species reannotated using syntenic alignments	Transcript isoform sequencing (TIF-seq), Ribosome Profiling	Most genes weakly constrained but a subset under strong selection, according to Neutrality Index, Direction of Selection, K_a_/K_s_, and McDonald-Kreitman tests	Subcellular localization demonstrated for five genes	~13,000 *de novo* genes	>65% of *de novo* transcripts are isoforms of ancient genes; >97% from TIF-seq dataset	[61]

## 2 Identification of *de novo* genes

### 2.1 Identification of *de novo* emerging sequences

There are two major approaches to the systematic identification of novel genes: genomic phylostratigraphy [46] and synteny-based methods. Both approaches are widely used, individually or in a complementary fashion (**Table 1**).

#### 2.1.1 Genomic phylostratigraphy

Genomic phylostratigraphy involves examining each gene in a focal species and inferring the presence or absence of ancestral homologs through the use of the BLAST sequence alignment algorithms [47] or related tools. Each gene in the focal species can be assigned an “age” (aka “conservation level” or “genomic phylostrata”) that is based on a predetermined phylogeny, with the age corresponding to the most distantly related species in which a homolog is detected [46]. When a gene lacks any detectable homolog outside of its own genome, or close relatives, it is said to be a novel, taxonomically-restricted or orphan gene, although such a designation is of course dependent on the group of species being searched against.

Phylogenetic trees are limited by the set of closely related genomes that are available, and results are dependent on BLAST search criteria [48]. Because it is based on sequence similarity, it is often difficult for phylostratigraphy to determine whether a novel gene has emerged *de novo* or has diverged from an ancestral gene beyond recognition, for instance following a duplication event. This was pointed out by a study that simulated the evolution of genes of equal age and found that distant orthologs can be undetectable for the most rapidly evolving genes [49]. When accounting for changes in the rate of evolution to portions of young genes that acquire selected functions, a phylostratigraphic approach was much more accurate at assigning gene ages in simulated data [50]. A subsequent pair of studies using simulated evolution found that phylostratigraphy failed to detect an ortholog in the most distantly related species for 13.9% of *D*. *melanogaster* genes and 11.4% of *S*. *cerevisiae* genes [51, 52]. Similarly, a spurious relationship between a gene’s age and its likelihood to be involved in a disease process was claimed to be detected in the simulated data [52]. However, a reanalysis of studies that used phylostratigraphy in yeast, fruit flies and humans found that even when accounting for such error rates and excluding difficult-to-stratify genes from the analyses, the qualitative conclusions were unaffected for all three studies [53]. The impact of phylostratigraphic bias on studies examining various features of *de novo* genes (see below) remains debated.

To increase the detectability of ancestral homologues, sensitive sequence-based similarity searches, such as CS-BLAST and Hidden Markov Model (HMM)-based searches, may also be used, alone or in combination with BLAST-based phylostratigraphy analysis, to identify *de novo* genes. The PSI-BLAST technique [54] is particularly useful for detecting ancient homologs. A benchmarking study found that some of these “profile-based” analyses were more accurate than conventional pairwise tools [55]. The impact of false positives, when genes are incorrectly inferred to have an ancestral homolog when they are new in reality, on our understanding of *de novo* gene birth has not yet been specifically assessed.

It is important to disentangle the technical difficulties associated with detection of the oldest ancestor of a gene, and estimates of how old a gene is (the ultimate goal of phylostratigraphy), from challenges linked to inferring the mechanisms by which a gene has evolved. Young and ancestral genes can all have evolved *de novo*, or through other mechanisms. The current approach of choice to determine whether a gene has emerged *de novo* is synteny, and can generally only be applied to young genes.

#### 2.1.2 Synteny-based approaches

Approaches based on the analysis of syntenic sequences in outgroups–blocks of sequence in which the order and relative positioning of features has been maintained–allow for the identification of non-genic ancestors of candidate *de novo* genes [6, 48]. Syntenic alignments are anchored by short, conserved “markers.” Genes are the most common marker in defining syntenic blocks, although k-mers and exons are also used [56, 57]. Assuming that a high-quality syntenic alignment can be obtained, confirmation that the syntenic region lacks coding potential in outgroup species allows a *de novo* origin to be asserted with higher confidence [48]. The strongest possible evidence for *de novo* emergence is the inference of the specific mutation(s) that created coding potential, typically through the analysis of microsyntenic regions of closely related species.

One challenge in applying synteny-based methods is the fact that synteny can be difficult to detect across longer timescales. To address this, various techniques have been tried, such as using exons clustered irrespective of their specific order to define syntenic blocks [57] or algorithms that use well-conserved genomic regions to expand microsyntenic blocks [58]. There are also difficulties associated with applying synteny-based approaches to genome assemblies that are fragmented [59] or in lineages with high rates of chromosomal rearrangements, as is common in insects [60]. Although synteny-based approaches have conventionally been lower-throughput in nature, they are now being applied to genome-wide surveys of *de novo* genes [44, 45, 61–66] and represent a promising area of algorithmic development for gene birth dating. Some have used synteny-based approaches in combination with similarity searches in an attempt to develop standardized, stringent pipelines [67] that can be applied to any group of genomes in an attempt to address discrepancies in the various lists of *de novo* genes that have been generated (see below).

### 2.2. Determination of *de novo* gene status

Even when the evolutionary origin of a particular sequence has been rigorously established computationally, it is important to note that there is a lack of consensus about what constitutes a genuine *de novo* gene birth event. One reason for this is a lack of agreement on whether or not the entirety of the newly genic sequence must be non-genic in origin. With respect to protein-coding *de novo* genes, it has been proposed that *de novo* genes be divided into subtypes corresponding to the proportion of the ORF in question that was derived from previously noncoding sequence [48]. Furthermore, for *de novo* gene birth to occur, the sequence in question must not just have emerged *de novo* but must in fact be a gene. Accordingly, the discovery of *de novo* gene birth has also led to a questioning of what constitutes a gene, with some models establishing a strict dichotomy between genic and non-genic sequences, and others proposing a more fluid continuum (see below). All definitions of genes are linked to the notion of function, as it is generally agreed that a genuine gene should encode a functional product, be it RNA or protein. There are, however, different views of what constitutes function, depending in part on whether a given sequence is assessed using genetic, biochemical, or evolutionary approaches [48, 68, 69].

It is generally accepted that a genuine *de novo* gene is expressed in at least some context [2], allowing selection to operate, and many studies use evidence of expression as an inclusion criterion in defining *de novo* genes. The expression of sequences at the mRNA level may be confirmed individually through conventional techniques such as quantitative PCR, or globally through more modern techniques such as RNA sequencing (RNA-seq). Similarly, expression at the protein level can be determined with high confidence for individual proteins using techniques such as mass spectrometry or western blotting, while ribosome profiling (Ribo-seq) provides a global survey of translation in a given sample. Ideally, to confirm that the gene in question arose *de novo*, a lack of expression of the syntenic region of outgroup species would also be demonstrated [70].

Confirmation of gene expression is only one approach to infer function. Genetic approaches, where one seeks to detect a specific phenotype or change in fitness upon disruption of a particular sequence, are considered by some to be the gold standard [69]; however, for large-scale analyses of entire genomes, obtaining such evidence is often not feasible. Other experimental approaches, including screens for protein-protein and/or genetic interactions, may also be employed to confirm a biological effect for a particular *de novo* ORF. As more is learned about a particular locus, standard molecular biology techniques can be applied to dissect its specific cellular role. Alternatively, evolutionary approaches may be employed to infer the existence of a molecular function from computationally-derived signatures of selection. In the case of TRGs, one common signature of selection is the ratio of nonsynonymous to synonymous substitutions (K_a_/K_s_ ratio), calculated from different species from the same taxon. This ratio indicates that the sequence in question is either evolving neutrally, or under either positive or negative selection. Evolutionary biologists tend to view only those sequences under selective constraint as being functional in the strict sense of the word [68]. Similarly, in the case of species-specific genes, polymorphism data may be used to calculate a pN/pS ratio from different strains or populations of the focal species. Given that young, species-specific *de novo* genes lack deep conservation by definition, detecting such signatures can be difficult without a large number of sequenced strains/populations. An example of this can be seen in *Mus musculus*, where three very young *de novo* genes lack signatures of selection despite well-demonstrated physiological roles [71]. Other signatures of selection, such as the degree of nucleotide divergence within syntenic regions, conservation of ORF boundaries, or for protein-coding genes, a coding score based on nucleotide hexamer frequencies, have instead been employed [72]. Despite these and other challenges in the identification of *de novo* gene birth events, there is now abundant evidence indicating that the phenomenon is not simply possible, but has occurred in every lineage systematically examined thus far.

## 3 Prevalence of *de novo* gene birth

### 3.1 Estimates of *de novo* gene numbers

Estimates regarding the frequency of *de novo* gene birth and the number of *de novo* genes in various lineages vary widely and are highly dependent on methodology. Studies may identify *de novo* genes by phylostratigraphy/BLAST-based methods alone, or may employ a combination of computational techniques (see above), and may or may not assess experimental evidence for expression and/or biological role. Furthermore, genome-scale analyses may consider all or most ORFs in the genome, or may instead limit their analysis to already annotated genes.

The *D*. *melanogaster* lineage is illustrative of these differing approaches. An early survey using a combination of BLAST searches performed on cDNA sequences along with manual searches and synteny information identified 72 new genes specific to *D*. *melanogaster* and 59 new genes specific to three of the four species in the *D*. *melanogaster* species complex. This report found that only 2/72 (~2.8%) of *D*. *melanogaster*-specific new genes and 7/59 (~11.9%) of new genes specific to the species complex were derived *de novo* [65], with the remainder arising via duplication/retroposition. Similarly, an analysis of 195 young (<35 million years old) *D*. *melanogaster* genes identified from syntenic alignments found that only 16 had arisen *de novo* [63]. In contrast, an analysis focused on transcriptomic data from the testes of six *D*. *melanogaster* strains identified 106 fixed and 142 segregating *de novo* genes [64]. For many of these, ancestral ORFs were identified but were not expressed. Highlighting the differences between inter- and intra-species comparisons, a study in natural *Saccharomyces paradoxus* populations found that the number of *de novo* polypeptides identified more than doubled when considering intra-species diversity [73]. In primates, one early study identified 270 orphan genes (unique to humans, chimpanzees, and macaques), of which 15 were thought to have originated *de novo* [44], while a later report identified 60 de novo genes in humans alone that are supported by transcriptional and proteomic evidence [66]. Studies in other lineages/organisms have also reached different conclusions with respect to the number of *de novo* genes present in each organism, as well as the specific sets of genes identified. A sample of these large-scale studies is described in **Table 1.**

A reanalysis of three such studies in murines that identified between 69 and 773 candidate *de novo* genes argued that the various estimates included many genes that were not in fact *de novo* genes [74]. Many candidates were excluded on the basis of no longer being annotated in the major databases. A conservative approach was applied to the remaining genes, which excluded candidates with paralogs, distantly related homologs or conserved domains, or that lacked syntenic sequence information in non-rodents. This approach validated ~40% of candidate *de novo* genes, resulting in an upper estimate of only 11.6 *de novo* genes formed (and retained) per million years, a rate ~5–10 times slower than what was estimated for novel genes formed by duplication [74]. It is notable that even after application of this stringent pipeline, the 152 validated *de novo* genes that remained still represents a significant fraction of the mouse genome likely to have originated *de novo*. Generally speaking, however, it remains debated whether duplication and divergence or *de novo* gene birth represent the dominant mechanism for the emergence of new genes [63, 65, 73, 75–77], in part due to the fact that *de novo* genes are likely both to emerge and to be lost more frequently than other young genes (see below).

### 3.2. Dynamics of *de novo* gene birth

It is important to distinguish between the frequency of *de novo* gene birth and the number of *de novo* genes in a given lineage. If *de novo* gene birth is frequent, it might be expected that genomes would tend to grow in their gene content over time; however, the gene content of genomes is usually relatively stable [6]. This implies that a frequent gene death process must balance *de novo* gene birth, and indeed, *de novo* genes are distinguished by their rapid turnover relative to established genes. In support of this notion, recently emerged *Drosophila* genes are much more likely to be lost, primarily through pseudogenization, with the youngest orphans being lost at the highest rate [78]; this despite the fact that some *Drosophila* orphan genes have been shown to rapidly become essential [63]. A similar trend of frequent loss among young gene families was observed in nematode genus *Pristionchus [79]*. In wild *S*. *paradoxus* populations, *de novo* ORFs emerge and are lost at similar rates [73]. Similarly, an analysis of five mammalian transcriptomes found that most ORFs in mice were either very old or species specific, implying frequent birth and death of *de novo* transcripts [77]. Nevertheless, there remains a positive correlation between the number of species-specific genes in a genome and the evolutionary distance from its most recent ancestor [80]. In addition to the birth and death of *de novo* genes at the level of the ORF, mutational and other processes also subject genomes to constant “transcriptional turnover”. One study in murines found that while all regions of the ancestral genome were transcribed at some point in at least one descendent, the portion of the genome under active transcription in a given strain or subspecies is subject to rapid change [81]. The “transcriptional turnover” of noncoding RNA genes is particularly fast as compared to that of coding genes [82].

## 4 Features of *de novo* genes

Recently emerged *de novo* genes differ from established genes in a number of ways. Across a broad range of species, young and/or taxonomically restricted genes or ORFs have been reported to be shorter in length than established genes, to evolve more rapidly, and to be less expressed [44, 75, 78, 79, 83–90]. Some of these reports, however, may have been partially influenced by the choice of homology-detection methods (see Genomic phylostratigraphy section). Their expression has also been found to be more tissue- or condition-specific than that of established genes [26, 28, 44, 64, 66, 75, 88, 91–93]. In particular, relatively high expression of *de novo* genes was observed in male reproductive tissues in *Drosophila*, mice, and humans (see below), and, in humans, in the cerebral cortex or the brain more generally [66, 94]. In animals with adaptive immune systems, higher expression in the brain and testes may at least in part be a function of the immune-privileged nature of these tissues. An analysis in mice found specific expression of intergenic transcripts in the thymus and spleen (in addition to the brain and testes), and it has been proposed that in vertebrates *de novo* transcripts must first be expressed in these tissues before they can be expressed in tissues subject to surveillance by immune cells [93].

### 4.1 Lineage-dependent features

Other general features of *de novo* genes appear dependent on the species or lineage being examined. This appears to partly be a result of the fact that genomes vary in their GC content, and young genes bear more similarity to non-genic sequences from the genome in which they arose than do established genes [95]. Features such as predicted intrinsic structural disorder (ISD), the percentage of transmembrane residues, and the relative frequency of various predicted secondary structural features all show a strong GC dependency in orphan genes, whereas in more ancient genes these features are only weakly influenced by GC content [95]. This is exemplified by the fact that in organisms with relatively high GC content, ranging from *D*. *melanogaster* to the parasite *Leishmania major*, young genes have high ISD [96, 97], while in a low GC genome such as budding yeast, young genes have low ISD [75, 83, 90, 95]. It is noteworthy, however, that the most ancestral budding yeast genes display smaller ISD than genes of intermediate age [75, 98].

### 4.2 Role of epigenetic modifications

An examination of *de novo* genes in *A*. *thaliana* found that they are both hypermethylated and generally depleted of histone modifications *[62].* In agreement with the proto-gene model (see below), methylation levels of *de novo* genes were intermediate between established genes and intergenic regions. The methylation patterns of these *de novo* genes are stably inherited, and methylation levels were highest, and most similar to established genes, in *de novo* genes with verified protein-coding ability [62]. In the pathogenic fungus *Magnaporthe oryzae*, less conserved genes tend to have methylation patterns associated with low levels of transcription [99]. A study in yeasts also found that *de novo* genes are enriched at recombination hotspots, which tend to be nucleosome-free regions [90].

In *Pristionchus pacificus*, orphan genes with confirmed expression display chromatin states that differ from those of similarly expressed established genes [89]. Orphan gene start sites have epigenetic signatures that are characteristic of enhancers, in contrast to conserved genes that exhibit classical promoters [89]. Many unexpressed orphan genes are decorated with repressive histone modifications, while a lack of such modifications facilitates transcription of an expressed subset of orphans, supporting the notion that open chromatin promotes the formation of novel genes [89].

## 5 Models and mechanisms of *de novo* gene birth

Several theoretical models and possible mechanisms of *de novo* gene birth have been described. The models are generally not mutually exclusive, and it is possible to imagine a number of plausible ways in which a *de novo* gene might emerge.

### 5.1 Order of events

#### 5.1.1 ORF first vs. transcription first

For birth of a *de novo* protein-coding gene to occur, a non-genic sequence must both be transcribed and acquire an ORF before becoming translated (**Fig 1A**). These events may in theory occur in either order, and there is evidence supporting both an “ORF first” and a “transcription first” model [2]. An analysis of *de novo* genes that are segregating in *D*. *melanogaster* with respect to their expression found that sequences that are transcribed had similar coding potential to the orthologous sequences from lines lacking evidence of transcription [64], supporting the notion that many ORFs, at least, exist prior to being expressed. The antifreeze glycoprotein gene *AFGP*, which emerged *de novo* in Arctic codfishes, provides a more definitive example in which the *de novo* emergence of the ORF was shown to precede that of the promoter region [100]. Furthermore, putatively non-genic ORFs long enough to encode functional peptides are numerous in eukaryotic genomes, and expected to occur at high frequency by chance [64, 75]. At the same time, transcription of eukaryotic genomes is far more extensive than previously thought, and documented examples also exist of genomic regions that were transcribed prior to the appearance of an ORF that became a *de novo* gene [101]. The proportion of *de novo* genes that are protein-coding is unknown, but the appearance of “transcription first” has led some to posit that protein-coding *de novo* genes may first exist as RNA gene intermediates. The case of bifunctional RNAs, which are both translated and function as RNA genes, shows that such a mechanism is plausible [102].

#### 5.1.2 “Out of Testis” hypothesis

An early case study of *de novo* gene birth, which identified five *de novo* genes in *D*. *melanogaster*, noted preferential expression of these genes in the testes [27], and several additional *de novo* genes were identified using transcriptomic data derived from the testes and male accessory glands of *D*. *yakuba* and *D. erecta* [26, 28] (see above). This was in keeping with the rapid evolution of genes related to reproduction that has been observed across a range of lineages [103–105], suggesting that sexual selection may play a key role in adaptive evolution and *de novo* gene birth. A subsequent large-scale analysis of six *D*. *melanogaster* strains identified 248 testis-expressed *de novo* genes, of which ~57% were not fixed [64]. It has been suggested that the large number of *de novo* genes with male-specific expression identified in *Drosophila* is likely due to the fact that such genes are preferentially retained relative to other *de novo* genes, for reasons that are not entirely clear [78]. Interestingly, two putative *de novo* genes in *Drosophila* (*Goddard* and *Saturn*) were shown to be required for normal male fertility [106].

In humans, a study that identified 60 human-specific *de novo* genes found that their average expression, as measured by RNA-seq, was highest in the testes [66]. Another study looking at mammalian-specific genes more generally also found enriched expression in the testes [107]. Transcription in mammalian testes is thought to be particularly promiscuous, due in part to elevated expression of the transcription machinery [108, 109] and an open chromatin environment [110]. Along with the immune-privileged nature of the testes (see above), this promiscuous transcription is thought to create the ideal conditions for the expression of non-genic sequences required for *de novo* gene birth. Testes-specific expression seems to be a general feature of all novel genes, as an analysis of *Drosophila* and vertebrate species found that young genes showed testes-biased expression regardless of their mechanism of origination [91].

### 5.2 Pervasive expression

With the development and wide use of technologies such as RNA-seq and Ribo-seq, eukaryotic genomes are now known to be pervasively transcribed [111–114] and translated [115]. Many ORFs that are either unannotated, or annotated as long non-coding RNAs (lncRNAs), are translated at some level, under at least some condition, or in a particular tissue [75, 115–118]. Though infrequent, these translation events expose non-genic sequence to selection. This pervasive expression forms the basis for several theoretical models describing *de novo* gene birth.

It has been speculated that the epigenetic landscape of *de novo* genes in the early stages of formation may be particularly variable between and among populations, resulting in variable levels of gene expression and thereby allowing young genes to explore the “expression landscape” [119]. The *QQS* gene in *A*. *thaliana* is one example of this phenomenon; its expression is negatively regulated by DNA methylation that, while heritable for several generations, varies widely in its levels both among natural accessions and within wild populations [119]. Epigenetics are also largely responsible for the permissive transcriptional environment in the testes, particularly through the incorporation into nucleosomes of non-canonical histone variants that are replaced by histone-like protamines during spermatogenesis [120].

#### 5.2.1 Proto-gene model

The proto-gene model proposes that de novo gene birth is mediated by a reservoir of “proto-genes” generated by pervasive expression of non-genic sequences [75]. It asserts that some of the proto-genes thereby exposed to the action of natural selection are occasionally retained and subsequently evolve the characteristics of genes. Proto-genes are thus expected to exhibit features intermediate between genes and non-genes. This model considers the genome as a spectrum ranging from non-genic to genic sequences, as opposed to the conventional binary classification scheme of gene vs. non-gene. The model makes use of the observation that in *S*. *cerevisiae*, several features of ORFs (see above) correlate with ORF age as determined by phylostratigraphic analysis [75]. A similar continuum with respect to gene age was seen for ORF features in a wide range of organisms (see above).

Most non-genic ORFs that are translated appear to be evolving neutrally [73, 75, 116]. The proto-gene model predicts, however, that expression of non-genic ORFs will occasionally provide an adaptive advantage to the cell. Adaptive proto-genes will gradually mature under selection, eventually leading to *de novo* gene birth. Differential translation of proto-genes in stress conditions, as well as an enrichment near proto-genes of binding sites for transcription factors involved in regulating stress response [75], support the adaptive potential of proto-genes. Furthermore, it is known that novel, functional proteins can be experimentally evolved from random amino acid sequences [121]. Random sequences are generally well-tolerated *in vivo*; many readily form secondary structures, and even highly disordered proteins may take on important biological roles [122–124]. The pervasive nature of translation suggests that new proto-genes emerge frequently, usually returning to the non-genic state.

Consistent with the notion that various features of ORFs exhibit a continuum that reflects their evolutionary age, a subsequent analysis, also in *S*. *cerevisiae*, found that ORF regulation by transcription factors, indicative of their integration into larger molecular networks, displays a similar continuum. Similarly, the likelihood of physical interactions, as well as the likelihood and strength of genetic interactions, is correlated with ORF age as determined by phylostratigraphy [125]. In contrast, with respect to certain predicted structural features such as β-strand content and aggregation propensity, the putative peptides encoded by proto-genes are similar to non-genic sequences and categorically distinct from canonical genes [125].

#### 5.2.2 Preadaptation model

The preadaptation model of *de novo* gene birth uses mathematical modeling to argue that when standing genetic variation that is normally hidden is exposed to weak or shielded selection, the resulting pool of “cryptic” variation is purged of “self-evidently deleterious” sequences, such as those prone to lead to protein aggregation, and enriched in potential adaptations relative to completely non-expressed sequences [126]. This revealing of cryptic variation and purging of deleterious non-genic sequences, which may be considered as proto-genes under the above model, is a byproduct of pervasive transcription and translation of intergenic sequences [118]. Beyond such purging, selection is thought to operate on non-genic sequences that already contain gene-like properties. Using the evolutionary definition of function (i.e. a gene is by definition under purifying selection), the preadaptation model asserts that “gene birth is a sudden transition to functionality [98]” that occurs as soon as an ORF acquires a selected effect. In contrast to the proto-gene model, recently emerged genes are expected to display exaggerated genic features, rather than features intermediate between old genes and non-genes [98]. In support of this, an analysis of ISD in mice found that young genes have higher ISD than old genes, while random non-genic sequences tend to show the lowest levels of ISD [98]. Although the observed trend may have partly resulted from a subset of young genes derived by overprinting [74], higher ISD in young genes was also seen among overlapping gene pairs [127]. Whether this trend holds over shorter timescales is debated [77, 128]. In wild *S*. *paradoxus* populations, ORFs with exaggerated gene-like features are found among the pool of translated intergenic polypeptides [73]. It is not clear whether such ORFs are preferentially retained.

The preadaptation model also proposes that in order to avoid the deleterious consequences associated with molecular errors, populations may either evolve local solutions, in which selection operates on each individual locus and a relatively high error rate is maintained, or global solutions that select for a low error rate and permit the accumulation of deleterious cryptic variation [126]. *De novo* gene birth is thought to be favored in populations that evolve local solutions, as the relatively high error rate will result in a pool of cryptic variation that is “preadapted” through the purging of deleterious sequences.

#### 5.2.3 Grow slow and moult model

The “grow slow and moult” model describes a potential mechanism of *de novo* gene birth, particular to protein-coding genes. In this scenario, existing protein-coding ORFs expand at their ends, especially their 3’ ends, leading to the creation of novel N- and C-terminal domains [129]. Novel C-terminal domains may first evolve under weak selection via occasional expression through read-through translation, as in the preadaptation model, only later becoming constitutively expressed through a mutation that disrupts the stop codon [126, 129]. Genes experiencing high translational readthrough tend to have intrinsically disordered C-termini [130]. Furthermore, existing genes are often close to repetitive sequences that encode disordered domains. These novel, disordered domains may initially confer some non-specific binding capability that becomes gradually refined by selection. Sequences encoding these novel domains may occasionally separate from their parent ORF, leading or contributing to the creation of a *de novo* gene [129]. Interestingly, an analysis of 32 insect genomes found that novel domains (i.e. those unique to insects) tend to evolve fairly neutrally, with only a few sites under positive selection, while their host proteins remain under purifying selection, suggesting that functional new domains emerge gradually and somewhat stochastically [131].

## 6 *De novo* gene birth and human health

In addition to its significance for the field of evolutionary biology, *de novo* gene birth has implications for human health. It has been speculated that novel genes, including *de novo* genes, may play an outsized role in species-specific traits [6, 37, 132]; however, many species-specific genes lack functional annotation [107]. Nevertheless, there is evidence to suggest that human-specific *de novo* genes are involved in disease processes such as cancer. *NYCM*, a *de novo* gene unique to humans and chimpanzees, regulates the pathogenesis of neuroblastomas in mouse models [133], and the primate-specific *PART1*, an lncRNA gene, has been identified as both a tumor suppressor and an oncogene in different contexts [44, 134, 135]. Several other human- or primate-specific *de novo* genes, including *PBOV1 [136]*, *GR6 [137, 138]*, *MYEOV [139]*, *ELFN1-AS1 [140],* and *CLLU1 [45]*, are also linked to cancer. Some have even suggested considering tumor-specifically expressed, evolutionary novel genes as their own class of genetic elements, noting that many such genes are under positive selection and may be neofunctionalized in the context of tumors [140].

The specific expression of many *de novo* genes in the human brain [66] also raises the intriguing possibility that *de novo* genes influence human cognitive traits. One such example is *FLJ33706*, a *de novo* gene that was identified in GWAS and linkage analyses for nicotine addiction and shows elevated expression in the brains of Alzheimer’s patients [141]. Generally speaking, expression of young, primate-specific genes is enriched in the fetal human brain relative to the expression of similarly young genes in the mouse brain [142]. Most of these young genes, several of which originated *de novo*, are expressed in the neocortex, which is thought to be responsible for many aspects of human-specific cognition. Many of these young genes show signatures of positive selection, and functional annotations indicate that they are involved in diverse molecular processes, and are specifically enriched for genes involved in transcriptional regulation relative to other functional classes [142].

In addition to their roles in cancer processes, *de novo* originated human genes have been implicated in the maintenance of pluripotency [143] and in immune function [44, 107, 144]. The preferential expression of *de novo* genes in the testes (see above) is also suggestive of a role in reproduction. Given that the function of many *de novo* human genes remains uncharacterized, it seems likely that an appreciation of their contribution to human health and development will continue to grow.

## Supporting information

S1 Text Version history of the text file. (XML)

S2 Text Peer reviews and response to reviews. (XML)
